# Circulating miR-21 and miR-181a as Biomarkers for Predicting Postoperative Complications Following Colorectal Cancer Resection: A Longitudinal Observational Study

**DOI:** 10.3390/jcm15041591

**Published:** 2026-02-18

**Authors:** Kornelija Rauduvytė, Marius Kryžauskas, Domas Drazdauskas, Vilius Ogaras, Paulina Kazlauskaitė, Sandra Ivanauskienė, Antanas Gulbinas, Tomas Poškus, Rasa Sabaliauskaitė, Agata Mlynska, Agnė Šeštokaitė, Rimantas Baušys, Matas Jakubauskas, Povilas Ignatavičius, Augustinas Baušys

**Affiliations:** 1 Laboratory of Experimental Surgery and Oncology, Translational Health Research Institute, Faculty of Medicine, Vilnius University, 08406 Vilnius, Lithuania; 2 Laboratory of Surgical Gastroenterology, Institute for Digestive Research, Medical Academy, Lithuanian University of Health Sciences, 44307 Kaunas, Lithuania; 3 Faculty of Medicine, Vilnius University, 03101 Vilnius, Lithuania; 4 Laboratory of Genetic Diagnostics, National Cancer Institute, 08406 Vilnius, Lithuania; 5 Faculty of Health Sciences, Klaipeda University, 92294 Klaipeda, Lithuania; 6 Laboratory of Immunology, National Cancer Institute, 08406 Vilnius, Lithuania; 7 Department of Chemistry and Bioengineering, Vilnius Gediminas Technical University, 10223 Vilnius, Lithuania; 8 Department of Surgery, Faculty of Medicine, Medical Academy, Lithuanian University of Health Sciences, 50161 Kaunas, Lithuania

**Keywords:** miR-21, miR-181a, colorectal cancer, postoperative complications, plasma microRNA

## Abstract

**Background/Objectives:** Early and accurate detection of postoperative complications (POCs) remains a major challenge in colorectal cancer (CRC) surgery, underscoring the need for reliable molecular biomarkers. This study evaluated whether plasma miR-21 and miR-181a can predict POCs following left-sided CRC resection. **Methods:** This longitudinal observational sub-study was conducted within a randomized controlled trial. Adult patients undergoing elective left-sided CRC resection were included. Plasma miR-21 and miR-181a levels were measured preoperatively and on postoperative day (POD) 6 using RT-qPCR. POCs were assessed according to Clavien–Dindo classification. Of 40 enrolled patients, 38 were included in the final analysis (15 with and 23 without postoperative complications). Discriminative performance was assessed using receiver operating characteristic analysis and correlations with inflammatory markers were evaluated. **Results:** No significant differences in plasma miR-21 or miR-181a levels were observed between groups at baseline or POD6 (all *p* > 0.05). Both biomarkers showed limited discriminative ability (AUC = 0.61 and 0.54, respectively), while a combined model of miR-181a + TNF-α improved performance (AUC = 0.76, 95% CI: 0.57, 0.94, *p* = 0.01). At baseline, miR-21 correlated strongly with miR-181a (ρ = 0.81, *p* < 0.001) and moderately inversely with TNF-α (ρ = −0.35, *p* = 0.043). **Conclusions:** MiR-21 or miR-181a measured at baseline or POD6 show limited predictive value for POCs after CRC surgery. Further studies would benefit from larger sample sizes and optimized sampling strategies that reflect possible early dynamic changes in these biomarkers.

## 1. Introduction

In 2022, colorectal cancer (CRC) was estimated to account for over 1.9 million new cases and 904,000 deaths [1]. Despite advances in chemotherapy and radiotherapy, surgical resection remains the primary curative treatment.

Postoperative complications (POCs) occur in approximately one-third of patients undergoing CRC surgery [2,3] and negatively affect overall survival, hospital stay duration, quality of life, and healthcare costs [4,5]. Conventional markers of inflammation, including C-reactive protein (CRP) and white blood cell (WBC) count, are clinically useful but lack reliability for the early detection of POCs in abdominal surgery [6]. Moreover, these markers typically increase only several days after surgery, limiting their early prognostic value [7]. To address these limitations, novel biomarkers such as microRNAs (miRNAs) are being explored as early predictors of POCs [8].

MiRNAs are short endogenous non-coding RNA molecules that regulate gene expression circulate extracellularly, acting both as biomarkers and mediators of intercellular communication [9]. As upstream regulators of gene expression [10], miRNAs may be able to reflect pathological alterations earlier than conventional inflammatory markers, providing a promising approach for early identification of POCs.

MiR-21 and miR-181a are known to have important roles in controlling inflammatory responses. MiR-21 is frequently upregulated in various cancers, where it modulates cell proliferation, migration, and invasion, and regulates the balance between the initial pro-inflammatory and the subsequent immunoregulatory phases [11]. MiR-181a plays an essential role in endothelial inflammation and regulates immune responses by directly targeting IL-1α and suppressing the production of pro-inflammatory mediators in monocytes [12].

Although miR-21 and miR-181a have been studied as biomarkers in CRC [13], sepsis [14], and POCs in cardiac and thoracic surgery [15,16], their potential role in predicting POCs following CRC surgery has not yet been investigated. To address this gap, we conducted a longitudinal observational study measuring plasma miR-21 and miR-181a levels preoperatively and on postoperative day (POD) 6 to evaluate their predictive value for POCs after CRC resection.

## 2. Material and Methods

### 2.1. Study Design and Ethics

This longitudinal observational study is a sub-study of two-arm randomized controlled trial (RCT) registered on ClinicalTrials.gov (NCT04013841, 10 July 2019). The results and methods of the original study have been published previously [17]. The study protocol received ethical approval from the Vilnius Regional Bioethics Committee (25 June 2019; No. 2019/6-1133-631). The research was carried out in accordance with the principles of the Declaration of Helsinki, and written informed consent was obtained from all participants before their inclusion in the study.

### 2.2. Study Setting and Participants

Patients aged 18 years or older who were scheduled for elective left-sided colorectal resection for CRC at the National Cancer Institute in Vilnius, Lithuania, between April and November 2021 were included. Exclusion criteria were (1) anticipated ileostomy; (2) known allergy to oral preparation agents; (3) planned multivisceral surgery; (4) emergency surgical patients; (5) history of inflammatory bowel disease; (6) prior gastrointestinal surgery compromising tract integrity; (7) clinical evidence of bowel obstruction due to tumor; and (8) pregnancy.

POCs were assessed independently by two surgical oncologists based on a comprehensive evaluation that included laboratory findings, radiological imaging, microbiological culture results, or intraoperative findings in cases requiring reoperation. Patients were categorized into two groups according to the occurrence of POCs: the POC+ group, which included patients who developed POCs, and the POC− group, comprising those who did not. A complication was classified as positive if the Clavien–Dindo grade was ≥ 1. Apart from the occurrence of POCs, all patients received the same standardized postoperative care protocol.

### 2.3. Study Outcomes

The study aimed to evaluate the prognostic value of circulating miR-21 and miR-181a in predicting POCs and to compare their performance with conventional inflammatory biomarkers, including CRP and WBC count. The discriminatory ability of each potential marker to distinguish patients with and without POCs was evaluated using receiver operating characteristic (ROC) curve analysis and the corresponding area under the curve (AUC) values.

### 2.4. Blood Sample Collection

Peripheral fresh blood samples were collected in EDTA tubes before surgery (baseline) and on POD6. Samples were centrifuged at 3000 rpm for 10 min at 4 °C (Heraeus Megafuge 8R, Thermo Fisher Scientific, Osterode am Harz, Germany), and the plasma fraction was carefully transferred into 1.5 mL tubes and immediately stored at −80 °C until further analysis.

### 2.5. Measurement of TNF-α

Serum TNF-α concentrations were measured in the same patient cohort as part of a previously conducted sub-study of the original RCT, as described previously [18]. The TNF-α values obtained in that investigation were used in the present analyses. TNF-α data were available for all included subjects.

### 2.6. Total RNA Purification

Total RNA was extracted from plasma using the miRNeasy Serum/Plasma Kit (QIAGEN, Hilden, Germany) following the manufacturer’s instructions. After lysis and incubation, 3.5 μL of 0.5 nM exogenous cel-miR-39-3p control was added to each sample. RNA purity and concentration were measured using a NanoDrop 2000 spectrophotometer (Thermo Fisher Scientific, Wilmington, DE, USA). Purified RNA was stored at −80 °C until further analysis.

### 2.7. cDNA Synthesis

Complementary DNA (cDNA) was synthesized from purified RNA using the TaqMan™ Advanced miRNA cDNA Synthesis Kit (Thermo Fisher Scientific, Waltham, MA, USA) according to the manufacturer’s instructions. The reactions were performed on a ProFlex™ 3 × 32–well PCR System (Applied Biosystems, Singapore). The resulting cDNA was either stored at −80 °C or immediately used for reverse-transcription quantitative polymerase chain reaction (RT-qPCR).

### 2.8. RT-qPCR

The levels of selected miRNAs (hsa-miR-21-5p and hsa-miR-181a-5p) were quantified by RT-qPCR using TaqMan Universal PCR Master Mix [2X] (Thermo Fisher Scientific, Vilnius, Lithuania) and TaqMan™ Human miRNA Assays (Applied Biosystems, Foster city, CA, USA). The following assay IDs were used: hsa-miR-21-5p (assay ID 477975), hsa-miR-181a-5p (assay I477857), and cel-miR-39-3p (assay ID 478293). Each reaction was performed in duplicate to assess technical variability, and no-template controls (without cDNA) were included to monitor potential contamination. RT-qPCR was performed on a QuantStudio™ 5 Real-Time PCR System (Thermo Fisher Scientific, Singapore) following the manufacturer’s protocol.

### 2.9. Statistical Analysis

All miRNA expression levels were normalized to the synthetic reference control cel-miR-39-3p using the 2^−ΔΔCt^ method. The resulting normalized values were used for all subsequent statistical analyses. Samples that yielded unreliable results due to technical qPCR errors were excluded from the final analysis.

Statistical analyses and data visualization were performed using RStudio v2025.9.1.401. Data normality was assessed using the Shapiro–Wilk test. Normally distributed data are presented as mean ± standard deviation (SD), while non-normally distributed data are presented as median (first quartile (Q1) to third quartile (Q3)). Categorical variables are presented as counts (n) and percentages (%) and compared using Fisher’s exact test or the χ^2^ test, while continuous variables were analyzed using paired t-tests or Mann–Whitney U tests, depending on data distribution. Correlations were assessed using Spearman’s rank correlation coefficient, with *p*-value < 0.05 indicating statistical significance.

ROC curve analysis was performed using the pROC package (version 1.19.0.1) in R to estimate the AUC and accuracy, specificity, sensitivity, positive predictive value (PPV), and negative predictive value (NPV) for each potential biomarker. DeLong’s test was used to compare AUCs between ROC curves derived from different combined biomarker panels [19].

This study is a sub-study conducted within a previous RCT; sample size and power calculations were reported in the parent trial, and no sample size calculation was performed for this sub-study [17].

## 3. Results

### 3.1. Baseline Characteristics

**Figure 1:**
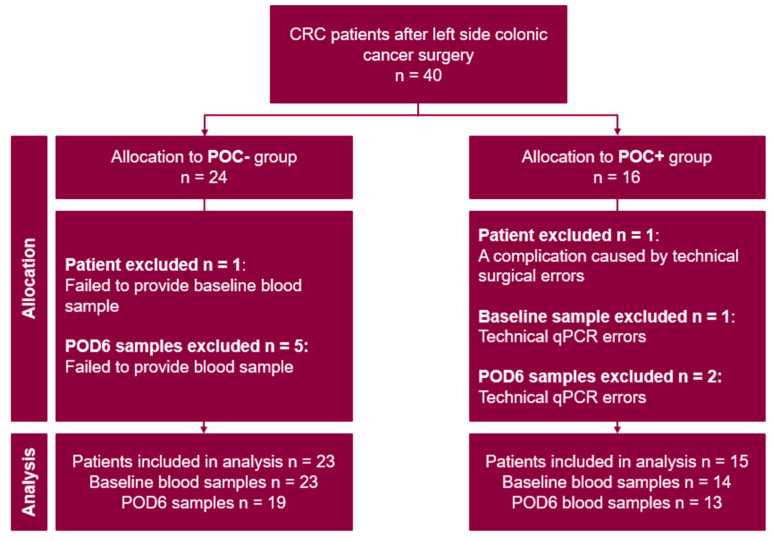
Flowchart of the analysis.

A total of 40 patients undergoing left-sided CRC surgery were enrolled in the study (Figure 1). Two (5%) patients were excluded from the final analysis.

In the POC−group (*n* = 24), one patient was excluded because both preoperative and POD6 blood samples were not provided. Additionally, five POD6 blood samples were not provided. In the POC+ group (n = 16), one patient was excluded due to a POC resulting from an iatrogenic injury. Additionally, one preoperative and two POD6 samples were excluded due to technical RT-qPCR errors.

In total, 38 patients were included in final analysis: 23 in the POC− group and 15 in the POC+ group. Among patients in the POC+ group, 6/15 (40%) developed wound suppuration, 3/15 (20%) had urinary tract infections, 2/15 (13%) developed intra-abdominal abscesses, 2/15 (13%) postoperative ileus, 1/15 (7%) anastomotic leakage, and 1/15 (7%) experienced other complications.

No significant differences were observed between the POC− and POC+ groups regarding demographic, clinicopathological, or treatment characteristics (Table 1).

### 3.2. Perioperative miRNA Levels in Patients Undergoing Colorectal Cancer Surgery

**Figure 2:**
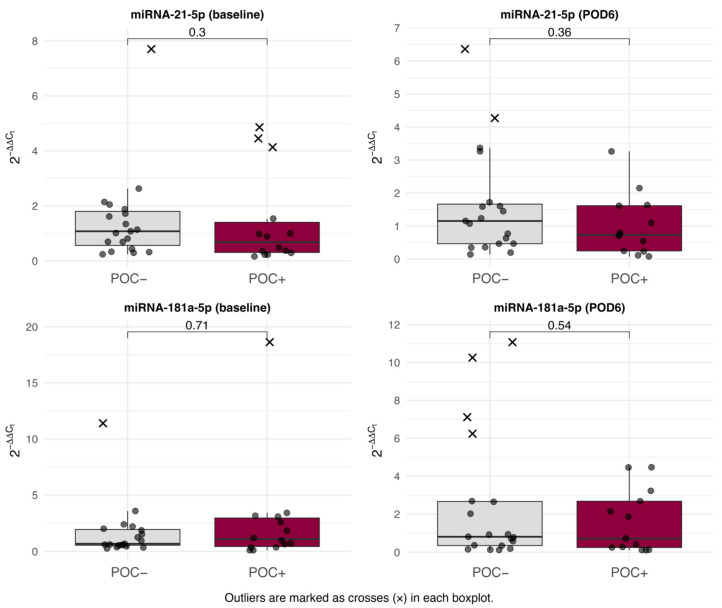
Circulating miR-21 and miR-181a in patients with or without postoperative complications (POCs) at baseline and postoperative day 6 (POD6). Dots represent individual patient data points and outliers are marked as black “x”.

There were no significant differences in relative circulating plasma miR-21 or miR-181a levels between the POC− and POC+ groups, either preoperatively or on POD6 (all *p* > 0.05) (Figure 2, Supplementary Table S1).

**Figure 3:**
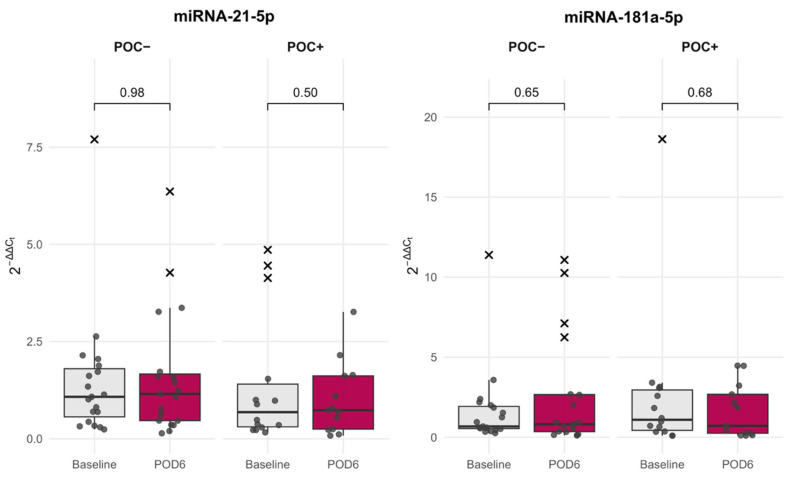
Perioperative inflammatory miRNA levels in patients with or without postoperative complications (POC). Dots represent individual patient data points and outliers are marked as black “x”. POD—postoperative day 6.

Comparisons within group also revealed no significant perioperative changes in either miRNA (Figure 3). Overall, both miRNAs demonstrated stable expression profiles across the perioperative period, with no distinct patterns differentiating patients who developed POCs from those who did not.

### 3.3. Perioperative Dynamics of CRP

**Figure 4:**
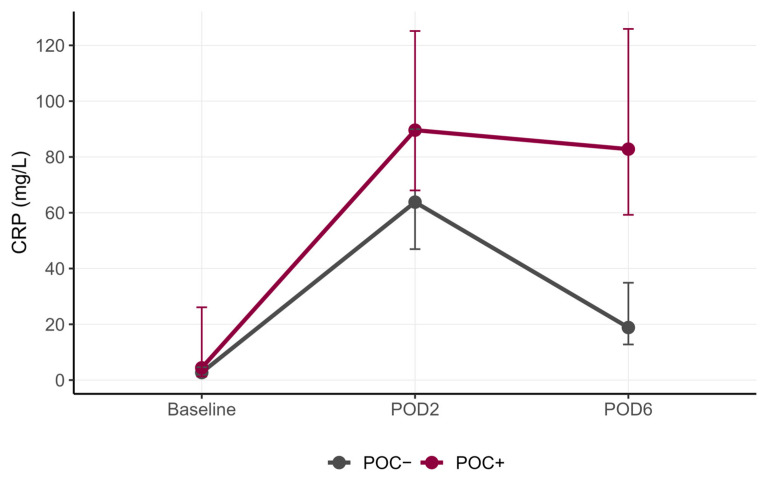
Perioperative C-reactive protein (CRP, mg/L) dynamics in patients with or without postoperative complications (POC). Data are presented as median (interquartile range) at baseline, postoperative day (POD) 2, and POD6.

To further characterize the postoperative inflammatory state, CRP levels were analyzed (Figure 4, Supplementary Table S2). On POD2, CRP levels were significantly higher in the POC+ group compared with the POC− group (median 89.6 mg/L vs. 63.8 mg/L, *p* = 0.011). By POD6, CRP levels declined in the POC− group, whereas in the POC+ group, they remained persistently elevated (median 18.8 mg/L vs. 82.8 mg/L, *p* < 0.001).

### 3.4. Baseline miRNA Levels as Predictors of Postoperative Complications

**Figure 5:**
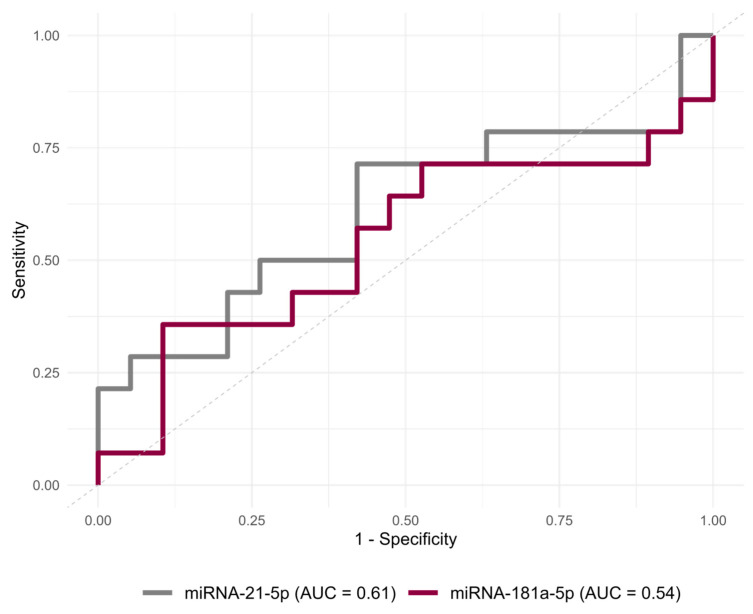
ROC curve analysis of baseline miR-21 and miR-181a to predict postoperative complications. AUC—area under the curve.

At baseline, neither miR-21 nor miR-181a demonstrated a significant ability to discriminate between patients who developed POCs and those who did not (Figure 5, Table 2). The AUC for miR-21 was 0.61 (95% CI: 0.40, 0.81, *p* = 0.24). Similarly, miR-181a showed an AUC of 0.54 (95% CI: 0.36, 0.76, *p* = 0.38). Comparison of ROC curves revealed no significant differences (all *p* > 0.05, DeLong’s test).

### 3.5. Correlation of Circulating miRNAs with Inflammatory Biomarkers

**Figure 6:**
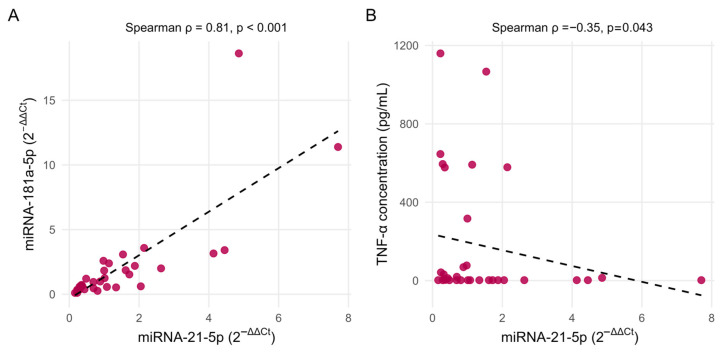
Correlation of relative circulating miRNAs with inflammatory indicators at baseline. (**A**) Correlation between plasma miR-21 and miR-181a levels. (**B**) Correlation between plasma miR-21 and TNF-α concentration. Dots represent individual patient data points and dashed lines indicate fitted linear trends.

To assess potential associations between circulating miRNAs and systemic inflammatory activity, baseline plasma levels of both miRNAs were correlated with established inflammatory biomarkers, including CRP, WBC count, and TNF-α (based on previous study findings [18]). A strong positive correlation was observed between miR-21 and miR-181a levels at baseline (Spearman’s ρ = 0.81, *p* < 0.001) (Figure 6A). A moderate negative correlation was found between miR-21 and TNF-α concentrations (Spearman’s ρ = −0.35, *p* = 0.043) (Figure 6B). No significant correlations were detected between miR-21 and either CRP or WBC count, nor between miR-181a and any of the evaluated inflammatory markers (all *p* > 0.05).

### 3.6. Combined Biomarker Models

**Figure 7:**
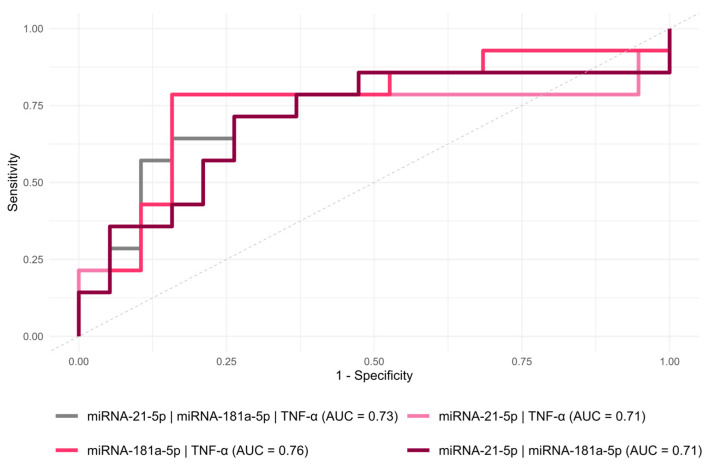
ROC curve analysis of combined inflammatory biomarker analysis to predict postoperative complications. AUC—area under the curve.

To evaluate whether combining circulating biomarkers could improve their diagnostic performance, several ROC models incorporating multiple baseline markers were tested. TNF-α was included in the combined analyses based on its previously reported association with POCs in the same patient cohort [18]. Among the combined biomarker models, miR-181a + TNF-α showed the highest AUC for predicting POCs (AUC = 0.76, 95% CI: 0.57, 0.94, *p* = 0.01) (Table 3, Figure 7). Comparison of ROC curves revealed no significant differences (all *p* > 0.05, DeLong’s test).

## 4. Discussion

This longitudinal observational study evaluated circulating plasma miR-21 and miR-181a as potential early biomarkers for predicting POCs following left-sided CRC resection. To the best of our knowledge, this study is the first to examine these specific miRNAs in this surgical context. Contrary to our initial hypothesis, based on studies showing miR-21 to predict pulmonary complications in thoracic surgery and miR-181a to be associated with postoperative muscle wasting in cardiac surgery [15,16], neither miR-21 nor miR-181a measured preoperatively or on POD6 was significantly associated with POCs.

Previous studies have implicated both miRNAs in inflammatory regulation and immune modulation. Upregulation of miR-21 has been linked to inflammatory activation and tumor progression in CRC [11,20]. Elevated circulating miR-21 has also been reported in other conditions associated with tissue injury or chronic inflammation, including vascular calcification in end-stage renal disease [21] and disease recurrence in CRC one to six months postoperatively [22]. Similarly, miRNA-181a modulates inflammatory signaling by targeting IL-1α, thereby suppressing proinflammatory cytokines such as TNF-α and IL-6 [23]. In CRC, higher miR-181a expression has been associated with aggressive tumor features and poorer prognosis [24]. In systemic inflammatory settings, such as sepsis, elevated miR-181a levels correlate with increased CRP, TNF-α, and procalcitonin, and worse survival [25].

In our study, both miRNAs exhibited stable perioperative expression, showing no significant difference between patients with or without POCs. This stability may indicate that these miRNAs are not dynamically responsive to inflammation at the selected time points, or that early transient peaks were missed. Circulating inflammatory miRNAs are known to change rapidly after surgical or ischemic stress, with miR-21 peaking within 24 h of injury [26,27]. By POD6, systemic inflammation may already be resolving, potentially masking early postoperative fluctuations. Indeed, miR-21 measured as early as two hours post-surgery has been predictive of postoperative pulmonary complications [15], while postoperative rises in miR-181a on POD2 have been associated with early muscle wasting after high-risk cardiothoracic surgery [16]. Future studies should therefore include serial postoperative sampling to better capture miRNA kinetics.

Despite the lack of perioperative changes, a strong positive correlation was observed between miR-21 and miR-181a, suggesting co-regulation through shared inflammatory or oncogenic pathways such as STAT3 [28,29,30]. Conversely, miR-21 demonstrated a moderate inverse correlation with TNF-α, which is a key proinflammatory cytokine [31]. Previous studies have reported inconsistent relationships, showing both positive [32] and weak negative correlations [33], underscoring the context-dependent nature of miRNA-mediated inflammatory regulation.

Although neither miR-21 nor miR-181a alone demonstrated predictive value, the combination of miR-181a with TNF-α modestly improved discriminative performance (AUC = 0.76, 95% CI: 0.57, 0.94, *p* = 0.01). However, the wide confidence interval underscores the uncertainty. Given that TNF-α has previously been shown to predict POCs in this cohort [18], the observed improvement in model performance is likely primarily driven by TNF-α. Therefore, the combined biomarker model should be interpreted strictly exploratory and hypothesis generating. Direct comparisons with similar CRC surgical cohorts are currently lacking, underscoring the need for further studies to validate multi-marker models combining inflammatory and molecular biomarkers in the postoperative setting. Future studies should explore such composite models alongside established inflammatory markers (e.g., CRP, IL-6) and assess dynamic miRNA fluctuations across the postoperative course.

Several limitations should be acknowledged. The analysis was based on an original RCT in which the sample size was predetermined for a primary outcome unrelated to this sub-study. Consequently, a separate power calculation for biomarker discovery or validation was not possible, and the study was conducted in an exploratory manner. A relatively small sample size reduced statistical power and generalizability, which is a common challenge in circulating miRNA studies, as both preanalytical and analytical variability can substantially affect detection and reproducibility [34,35]. Notably, previous studies reporting statistically significant results in surgical complication prediction included 42 [16] and 368 [15] participants, highlighting that larger cohorts may be required to achieve robust and reproducible findings. In addition, POCs were classified as Clavien–Dindo ≥1, including both minor deviations and clinically significant events. Such heterogeneity may have reduced the sensitivity to detect biomarker associations specific to more severe and clinically relevant events. Regarding methodological considerations, spike-in-based normalization does not account for factors such as hemolysis or platelet contamination, which may influence circulating miRNA levels. Furthermore, sampling at only two time points may have missed transient perioperative miRNA fluctuations. Although POD6 is clinically relevant in the postoperative course, as supported by persistently elevated CRP levels in our cohort, it may not have been the optimal time point to capture peak miRNA dynamics. Moreover, due to technical limitations inherent to plasma-based miRNA quantification, a better approach for future studies is to assess it by conducting Next Generation Sequencing so as to obtain high-throughput results. Furthermore, postoperative inflammation is a complex and multifactorial process driven by surgical trauma, ischemia–reperfusion injury, anesthesia, and microbial translocation [15,36], each of which may trigger distinct yet overlapping miRNA responses. Additionally, miRNA changes may reflect the underlying cancer itself [13,20,22], and the surgical procedure may not induce additional substantial alterations. Lastly, it should be noted that the study included only left-sided CRC resection, as this was a sub-study of a previous RCT. Such selection bias may preclude generalizability of our findings to other high-risk CRC surgical procedures, including right-sided resections and emergency surgery.

Despite these limitations, this study provides novel data on perioperative miR-21 and miR-181a expression in CRC surgery and highlights the potential of combined molecular–inflammatory biomarker models. Larger, longitudinal studies with optimized sampling strategies should be performed to clarify the dynamic role of circulating miRNAs in postoperative inflammation and complication risk.

## 5. Conclusions

In summary, this study found no significant association between perioperative plasma miR-21 or miR-181a levels and POC occurrence among surgical left-side CRC patients. These findings suggest a limited utility of these biomarkers for postoperative risk prediction. Future studies should explore larger cohorts, multi-marker approaches, and optimized sampling strategies to better capture dynamic postoperative changes.

## Figures and Tables

**Table 1:** Baseline and treatment characteristics of patients with versus without postoperative complications.

Characteristic	POC− N = 23 ^1^	POC+ N = 15 ^1^	*p*-Value
Age	67 (53, 70)	72 (59, 77)	0.147
Gender			0.126
Female	8 (Export Date: 16 January 2026; Cited By: 61.8)	9 (60.0)	
Male	15 (65.2)	6 (40.0)	
Smoking status			>0.999
Non-smokers	21 (91.3)	14 (93.3)	
Smokers/ex-smokers	2 (8.7)	1 (6.7)	
Alcohol consumption			0.944
Drinkers	11 (47.8)	7 (46.7)	
Non-drinkers	12 (52.2)	8 (53.3)	
Bowel preparation			0.740
Oral preparation	12 (52.2)	7 (46.7)	
Rectal enema	11 (47.8)	8 (53.3)	
pT			0.337
T1-2	8 (34.8)	2 (13.3)	
T3-4	13 (56.5)	12 (80.0)	
Not available	2 (8.7)	1 (6.7)	
pN			0.531
N0	15 (65.2)	7 (46.7)	
N1-2	6 (26.1)	7 (46.7)	
Not available	2 (8.7)	1 (6.7)	
pM			0.264
M0	21 (91.3)	12 (80.0)	
M1	0 (0.0)	2 (13.3)	
Not available	2 (8.7)	1 (6.7)	
Clinical stage			0.499
1	10 (43.5)	4 (26.7)	
2	4 (17.4)	2 (13.3)	
3	9 (39.1)	9 (60.0)	
Pathological stage			0.234
I-II	15 (65.2)	6 (40.0)	
III-IV	6 (26.1)	8 (53.3)	
Not available	2 (8.7)	1 (6.7)	
Surgical approach			0.285
Laparoscopic	18 (78.3)	9 (60.0)	
Open	5 (21.7)	6 (40.0)	
ASA			0.188
1-2	21 (91.3)	11 (73.3)	
>2	2 (8.7)	4 (26.7)	
CCI			0.115
1-5	20 (87.0)	9 (60.0)	
>5	3 (13.0)	6 (40.0)	
Length of surgery, minutes	115 (95, 150)	145 (100, 155)	0.094
Tumor localization			0.848
Sigmoid colon	13 (56.5)	8 (53.3)	
Rectum	9 (39.1)	7 (46.7)	
Not available	1 (4.3)	0 (0.0)	
White blood cell count, ×10^9^/L	6.9 (5.5, 8.8)	7.0 (6.3, 8.5)	0.869
C-reactive protein, mg/L	3 (2, 5)	4 (1, 26)	0.420
Neoadjuvant therapy	0 (0)	0 (0)	

^1^ Median (Q1, Q3) or n (%); pT—pathological tumor stage; pN—pathological nodal stage; pM—pathological distant metastasis according to TNM classification; ASA—American Society of Anesthesiologists physical status; CCI—Charlson Comorbidity Index.

**Table 2:** Diagnostic performance of baseline relative circulating miRNAs for postoperative complications.

	AUC (95% CI)	Sensitivity	Specificity	PPV	NPV	Accuracy
miR-21	0.61 (0.40, 0.82)	0.71	0.58	0.56	0.73	0.64
miR-181a	0.54 (0.32, 0.76)	0.36	0.89	0.71	0.65	0.67

AUC—area under the curve; CI—confidence interval; PPV—positive predictive value; NPV—negative predictive value.

**Table 3:** Diagnostic performance of combined baseline biomarkers for postoperative complications.

	AUC (95% CI)	Sensitivity	Specificity	PPV	NPV	Accuracy
miR-21 + miR-181a	0.71 (0.51, 0.91)	0.71	0.74	0.67	0.78	0.73
miR-21 + miR-181a + TNF-α	0.73 (0.53, 0.93)	0.64	0.84	0.75	0.76	0.76
miR-21 + TNF-α	0.71 (0.50, 0.93)	0.79	0.84	0.79	0.84	0.82
miR-181a + TNF-α	0.76 (0.57, 0.94)	0.79	0.84	0.79	0.84	0.82

AUC—area under the curve; CI—confidence interval; PPV—positive predictive value; NPV—negative predictive value.

## Data Availability

The data presented in this study are available on request from the corresponding author due to privacy and ethical restrictions.

## Supplementary Materials

The following supporting information can be downloaded at https://www.mdpi.com/article/10.3390/jcm15041591/s1, Supplementary Table S1: Median (Q1, Q3) levels of miRNA-21 and miRNA-181a at baseline and postoperative day 6 in patients with (POC+) and without (POC–) postoperative complications (POC); Supplementary Table S2: Perioperative C-reactive protein (CRP, mg/L) dynamics in patients with or without postoperative complications (POC). Data are presented as median (interquartile range) at baseline, postoperative day (POD) 2, and POD6. Group comparisons were performed using the Mann-Whitney U test.
