# Supplementary material for: Circulating miR-21 and miR-181a as Biomarkers for Predicting Postoperative Complications Following Colorectal Cancer Resection: A Longitudinal Observational Study
Source: J Clin Med. 2026 Feb 18;15(4):1591. doi: 10.3390/jcm15041591 (PMC12941494; doi:10.3390/jcm15041591)
Supplement: Supplementary file 1 [file jcm-15-01591-s001.zip › jcm-4133918-supplementary.pdf]

Journal of
Clinical Medicine

MDPI

Table S1. Median (Q1, Q3) levels of miRNA-21 and miRNA-181a at baseline and postoperative day 6 in patients with (POC+) and without (POC–) postoperative complications (POC).

	**POC -<br>n = 15**	**POC +<br>n = 23**
Baseline		
MiR-21	1.1 (0.6, 1.8)	0.9 (0.3, 2.8)
MiR-181a	0.7 (0.5, 1.9)	1.2 (0.5, 3.1)
Postoperative day 6		
MiR-21	1.2 (0.5, 1.7)	0.8 (0.4, 1.9)
MiR-181a	0.8 (0.4, 2.7)	1.9 (0.3, 3.8)

Table S2. Perioperative C-reactive protein (CRP, mg/L) dynamics in patients with or without post-operative complications (POC). Data are presented as median (interquartile range) at baseline, post-operative day (POD) 2, and POD6. Group comparisons were performed using the Mann-Whitney U test.

	**Timepoint**	**POC -<br>n = 15**	**POC +<br>n = 23**	***p* value**
C-reactive protein, mg/L, Median (Q1, Q3)	***Baseline***	2.7 (1.8, 4.6)	4.4 (1.3, 26.1)	0.420
	POD2	63.8 (47.0, 90.0)	89.6 (68.0, 125.0)	**0.011**
	POD6	18.8 (12.8, 34.9)	82.8 (59.2, 126.0	**< 0.001**
